# An enhanced-performance multisensing progressive cellular μGC: design advances and blind test results

**DOI:** 10.1038/s41378-025-00984-6

**Published:** 2025-07-11

**Authors:** Declan Winship, Weilin Liao, Hsueh-Tsung Lu, Irene Lara-Ibeas, Xiangyu Zhao, Qu Xu, Tao Qian, Robert Gordenker, Yutao Qin, Yogesh B. Gianchandani

**Affiliations:** 1 Department of Electrical Engineering and Computer Science, and Center for Wireless Integrated MicroSensing and Systems (WIMS²), University of Michigan, Ann Arbor, MI 48109 USA; 2 Department of Mechanical Engineering, and Center for Wireless Integrated MicroSensing and Systems (WIMS²), University of Michigan, Ann Arbor, MI 48109 USA; 3 Department of Integrative Systems + Design, and Center for Wireless Integrated MicroSensing and Systems (WIMS²), University of Michigan, Ann Arbor, MI 48109 USA

**Keywords:** Engineering, Chemistry

## Abstract

Many environmental, industrial, and security applications demand in-field analysis of chemical vapors. Whereas microscale gas chromatographs (µGCs) are promising candidates, reliable in-field chemical analysis particularly demands repeatability, humidity tolerance, and in-field reference. Using a µGC with substantial monolithic integration (of preconcentrators, separation columns, and capacitive and photoionization detectors), this paper reports chip-level and system-level advancements towards reliable chemical analysis. Thermal management is advanced using tailored heater designs to compensate for boundary conditions and cooling. Fence electrodes are incorporated into on-chip photoionization detectors, reducing responses due to humidity by >98%. The repeatability of retention time is advanced by introducing closed-loop flow control, reducing the relative standard deviation of retention time to only 0.29–0.43%, which represents a 4–5× improvement over open-loop flow control. A miniature reservoir for a chemical reference standard is also incorporated on board, providing the ability to correct for drifts in retention time and the ability to directly measure retention times relative to the reference chemical. A set of blind false alarm tests was performed for fixed target analytes in the presence of partially coeluting interferent species. A separate set of blind chemical recognition tests was also performed for various analytes of concealed identities. Overall, the results were largely successful and showed the promise of the reported µGC instrument and modules for broad chemical screening and long-term in-field deployment.

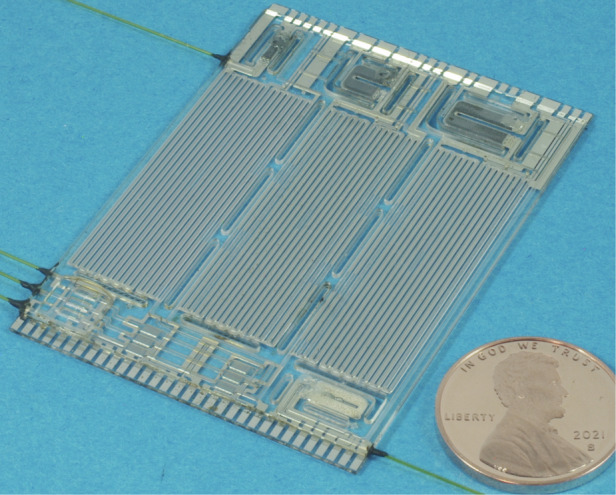

## Introduction

With the prospects of in-field analysis of complex chemical vapor compositions, microscale gas chromatographs (μGCs) have been investigated since the 1970s^1,2^. A μGC aims to separate the mixed analytes in a collected sample into individual species. The separation is performed by passing the mixed analytes through a separation column, which is a channel that provides partitioning of the analytes between a coated stationary phase material and the carrier gas^3,4^. Each analyte elutes from the column as a concentrated peak with a characteristic retention time, which is the primary parameter by which analyte species are recognized. The concentration peaks are typically detected in a non-specific manner using thermal conductivity or other detectors that are not necessarily selective^5,6^. Analyte recognition can be enhanced by using multiple detectors that are partially selective but complementary^7–9^, or by a spectrometer that provides very high selectivity^10,11^. For analytes at low concentrations, μGCs typically incorporate preconcentrators that accumulate the sample over time and subsequently inject the sample into the separation column^12,13^. Additional components may include valves, pumps, and sometimes a carrier gas bottle. These components, either custom-designed and microfabricated for these works, or sourced from commercial suppliers of miniature parts, have been combined into complete systems of μGCs^7,11,14–16^.

Our group has previously reported a μGC that implements a multisensing progressive cellular architecture (MPCA) with substantial monolithic integration^9^. This architecture incorporates three μGC cells on a monolithic chip, with each cell customized for a different volatility range of target analytes. Each cell is comprised of a preconcentrator, a separation column, and multiple detectors; the preconcentrator material and separation column coating provide the customization. The system is operated in two steps: the sampling step and the analysis step. During the sampling step, the analyte sample sequentially passes through the three preconcentrators that are arranged in the order of increasing adsorption strength. As a result, preliminary sorting of analytes is achieved: low volatility analytes are trapped in the upstream preconcentrator, whereas higher volatility analytes break through into the downstream preconcentrators. During the subsequent analysis step, performed sequentially for the three cells, the sample trapped in each cellular preconcentrator is desorbed and injected into the corresponding separation column. Each column is tailored to its analyte volatility range, allowing for much lower column temperatures than a unicellular design with the same range. Each cell incorporates two complementary capacitive detectors (CapDets) and a photoionization detector (PID). A single vacuum ultraviolet (VUV) lamp is shared by the PIDs from all three cells, which are collectively named the arrayed integrated photoionization detectors (AiPDs). The responses of the three detectors in each cell form a detector response pattern (DRP), which comprises peak height ratios between the three detectors.

A μGC typically requires different temperature controls for its different components, leading to two requirements: uniform heating within each component and thermal isolation between components. For a monolithic μGC chip, a silicon substrate is thermally conductive and provides good temperature uniformity, but it poses challenges with thermal isolation^17–19^. In contrast, a thermally non-conductive glass or fused silica substrate^9,20^ facilitates thermal isolation between components, but the heating uniformity requires attention^9^.

Another aspect is the humidity tolerance of the on-chip PIDs. As a widely used detector for μGCs^14,16,21–23^, the PID uses VUV radiation to ionize analytes, then collects the electric current resulting from ion-electron pairs using a pair of electrodes. PIDs may suffer parasitic surface currents due to humidity present in the vapor sample^24–26^. In commercial PIDs, a third electrode (i.e., the fence electrode) is typically incorporated to block surface currents^25–27^. However, in previously reported microfabricated PIDs^9,14,22,23^, the fence electrode has not been incorporated.

The third aspect is the carrier gas flow rate repeatability, which directly affects the repeatability of the analyte retention time. For many μGCs that use a pressurized carrier gas bottle^7,17,28,29^, the flow rate repeatability relies on a regulator for the bottle. As the gas bottles are sizable consumables limiting miniaturization and long-term field deployment, other μGCs use filtered ambient air as the carrier gas^30,31^. However, this ambient air is driven by miniature pumps, and by default, commercial miniature pumps operate in an open-loop manner without any specification on the flow rate repeatability.

The fourth aspect is the availability of an internal chemical reference, which is a subject rarely reported for µGCs. For long-term field deployment, a built-in chemical reference enables in situ calibration or verification of the µGC performance. While commercial products are already available for those GCs operated in a laboratory environment, these products might not be amenable to field-deployed µGCs. For example, the compressed lecture bottles storing vapor phase reference chemicals are relatively bulky. The permeation tubes contain reference chemicals that permeate through a polymeric membrane and mix with a flow towards the instrument under calibration^32^; the delivery of accurate concentration requires accurate temperature control, flow rate control, and pre-calibration of the permeation rate. Microfabricated reservoirs containing liquid phase reference chemicals have been reported^33,34^. In a transported and field-deployed instrument, the liquid phase reference chemicals may evaporate from the reservoir and re-condense elsewhere, which may cause liquid chemicals to be distributed at undesired locations.

**Fig. 1:**
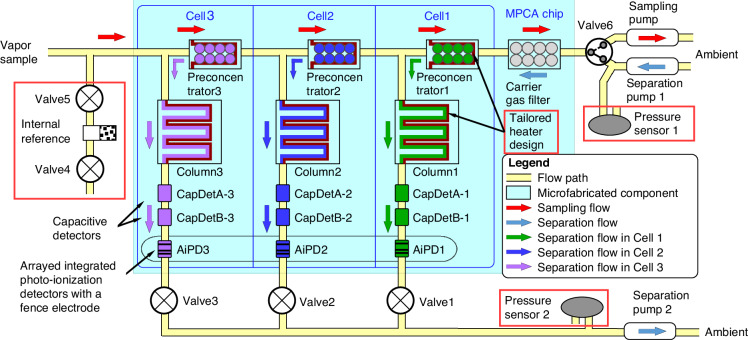
Fluidic architecture of the MPCA2 system, which includes the MPCA2 chip, an internal reference, commercial microvalves, two separation pumps, and one sampling pump, and pressure sensors for closed-loop flow control. The red boxes indicate key reliability features discussed in this work

Corresponding to the four aspects above, this paper reports a μGC with an MPCA that demonstrates advancements toward reliability and precision, which assist chemical recognition. This μGC (Fig. 1), named the MPCA2, addresses the aforementioned challenges by implementing the following: (1) component-wise uniform heating on a thermally non-conductive substrate that monolithically integrates multiple components; (2) integrated fence electrodes for the on-chip PIDs; (3) closed-loop control of the carrier gas flow rate by using pressure sensors in conjunction with the pumps; (4) an easily configurable miniature internal chemical reference based on partition equilibrium without unconfined liquids. Although these advancements are demonstrated in the context of an MPCA system in this work, they may benefit other μGCs as well.

It is worth noting that experimental assessments reported in this work lean toward the reliability and precision aspects of system performance, as well as challenging blind tests. Some aspects of the MPCA2 system performance are similar to the MPCA system that we previously reported^9^; these include, for example, response linearity, carry-over characteristics, chromatographic resolution, DRP characteristics, and sensitivity. As these general aspects have been previously reported^9^, they are minimally addressed or not repeated in this work.

## Design and implementation

For advancing reliability and chemical recognition capabilities, both chip-level and system-level enhancements are implemented. At the chip level, compensatory heater designs and PIDs with fence electrodes are implemented on a monolithically integrated MPCA2 chip (section “MPCA2 chip design”). At the system level, the closed-loop flow control and chemical reference are implemented along with the MPCA2 chip in a larger MPCA2 system (section “System architecture and enhancements”).

### MPCA2 chip design

#### Chip structure

**Fig. 2:**
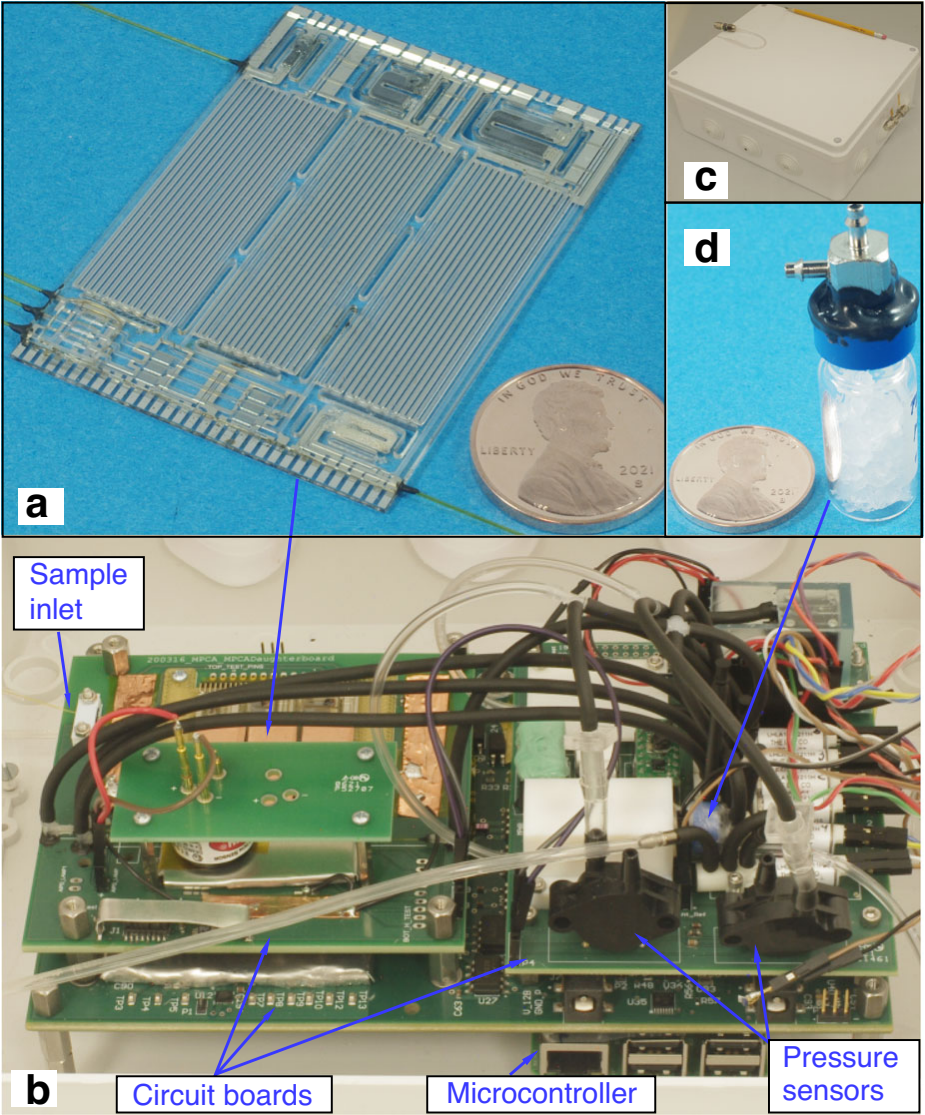
Photographs of the MPCA2 components and system. **a** The MPCA2 chip. **b** The system interior. **c** The enclosure of the system and **d** the internal reference

The MPCA2 chip monolithically integrates three preconcentrators, three separation columns, three CapDets that provide only positive peaks (i.e., CapDetAs), three CapDets that provide both positive and negative peaks (i.e., CapDetBs), three AiPDs, and a carrier gas filter. The three preconcentrators are designed with different sizes and different sorbent materials (Table 1) for adsorbing different volatility ranges of chemicals. The three separation columns adopt the same channel design (0.57 m long, 150 μm deep, and 500 μm wide), the same footprint (35 × 12.5 mm^2^), the same stationary phase coating material (polydimethylsiloxane, i.e., PDMS, Part # OV-1-LV, Ohio Valley Specialty Company, OH, USA), but different stationary phase coating thicknesses (Table 1) for separating different volatility ranges of chemicals. Each CapDet is a PDMS-coated capacitor, which incorporates interdigitated thin-film electrodes of 1.5 μm width and 1.5 μm gaps over an area of 1.7 mm^2^. The coating thicknesses are 0.4 μm for all the CapDetAs and 2 μm for all the CapDetBs. The AiPD design is described in the “AiPD fence electrode” subsection. The carrier gas filter is structurally similar to the preconcentrators, but is packed with Molecular Sieve 5A (Sigma Aldrich, MO, USA) that adsorbs moisture and thus provides dried air for separation and for purging. It can be regenerated in situ by the combination of heating and gas flow, provided by an on-chip heater and a sampling pump (Fig. 1 and section “System architecture and enhancements”), respectively. The footprint of the MPCA2 chip is 40.3 × 55.7 mm^2^ (Fig. 2a). Figure 2 also shows circuit boards supporting the MPCA2 chip, fluidic connections, and pressure sensors for closed-loop flow control (Fig. 2b), and an internal chemical reference (Fig. 2d), all housed in an enclosure (Fig. 2c).

**Table 1:** Cell-dependent design parameters in the MPCA2 chip

Cell name	Cell1	Cell2	Cell3
Preconcentrator sorbent type	Carboxen® 1003	Carbopack^TM^ X	Carbograph^TM^ 2
Sorbent surface area (m^2^/g)	1000	240	10
Sorbent chamber volume (μL)	15.2	8.6	2.7
Estimated sorbent mass (mg)	7.4	3.5	1.8
Column coating thickness (μm)	10	5	0.5

The MPCA2 chip consists of a stack of two bonded fused silica dies. A 675 μm-thick top die provides sandblasted gas flow channels and sorbent chambers at 180 and 500 μm depths, respectively. A 500 μm-thick bottom die contains 150–300 nm thick Ti/Pt patterns, which provide heaters, thermistors, and detector electrodes. Both dies contain sandblasted through-substrate cutouts for thermal isolation. For the AiPDs, the top die further incorporates upper openings that are sealed by an MgF_2_ window, which is transparent to VUV radiation. The bonding and sealing between all these fused silica dies and MgF_2_ window are provided by an epoxy (Epotek-377, Epoxy Technology Inc., MA, USA).

#### Heater design and modeling

**Fig. 3:**
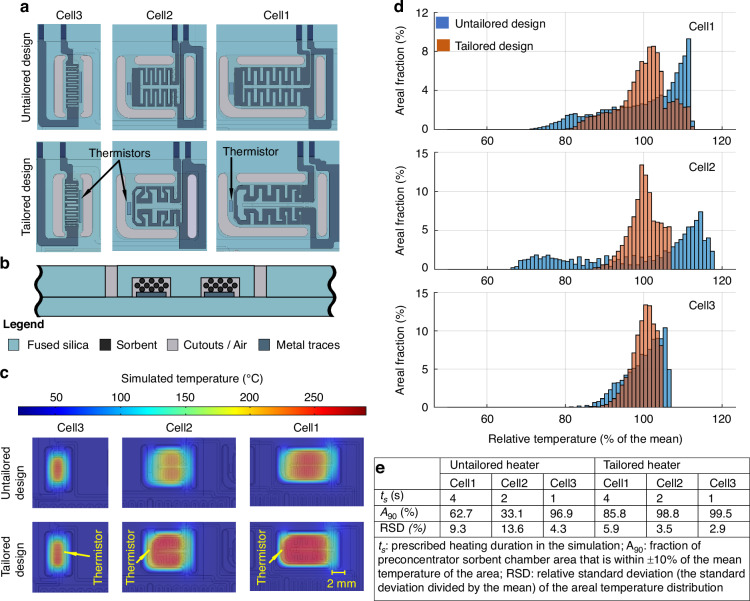
Preconcentrator heater layout designs and simulation results. **a** Untailored heater design and tailored heater design layouts. **b** Illustration of preconcentrator cross-section. **c** Comparison of simulated temperature distributions at the end of heating between untailored and tailored heater designs for the three preconcentrators. **d** Statistics of simulated temperature distribution over preconcentrator sorbent chamber area for untailored and tailored designs in each preconcentrator when heated for the prescribed durations. **e** Summary of the simulated temperature distribution nonuniformity

Because the substrate of the monolithic chip is comprised of fused silica, which has low thermal conductivity, the edge cooling effects are manifest as nonuniform temperature distribution over an area, even if it has a uniform supply of Joule heating power. More specifically, compared to the center area of a heated component, its perimeter and physical connections to other unheated components are typically more dissipative and hence colder. This problem can be addressed by a compensatory heater layout that is geometrically tailored to provide larger power density to the more dissipative areas. Using this strategy, the MPCA2 preconcentrator heaters are designed as serpentine traces with long and narrow segments near the perimeter of the preconcentrator, but with wider and shorter segments near the center (Fig. 3a, b). Certain segments are shaped and curved for corner compensation based on simulations.

To evaluate the heating uniformity, the MPCA2 chip is modeled using a finite element analysis (FEA) built in COMSOL Multiphysics 5.3. The model studies the transient thermal responses of the heated components by coupling electric current, Joule heating, heat conduction and convection, and the temperature coefficient of resistance (TCR). The model is constructed to be consistent with the actual implementation, where the µGC chip is seated on an Ultem 1010 substrate, which is further mounted on a PCB. The Ultem 1010 substrate incorporates an array of pillars, which create an air gap underneath the MPCA2 chip to reduce thermal dissipation into the underlying circuit board while providing mechanical support to the chip. The electrical resistivity and TCR values used in the model are obtained from experimentally measured results and have values of 3.8 × 10^−7^ Ω∙m and 0.003 °C^−1^ at 20 °C, respectively (section S1). The sorbent beds of the preconcentrators are approximated as a solid material with a thermal conductivity of 6 W/mK^35^. Ambient temperature is assumed to be 20 °C, and natural air convection is assumed on all exterior surfaces.

In actual system operation, the preconcentrator temperature is servo-controlled with the thermistor providing the temperature readout. In this closed loop, the preconcentrator heater is initially subject to 24 V, which is the largest heater voltage available from the system circuitry. After a few seconds, as the measured temperature approaches the target temperature, the heater voltage is decreased to maintain the target temperature. To simplify the simulation, this closed-loop control is not simulated. Instead, in the simulation, the heater is supplied by a constant 24 V, and the temperature distribution is inspected at the time when the preconcentrator has been heated for the prescribed duration (4 s in Cell1, 2 s in Cell2, and 1 s in Cell3), thus providing an evaluation that is approximate but nevertheless representative of the uniformity of temperature in the preconcentrator.

The temperature distribution obtained from the simulation is shown in Fig. 3c. In this, the preconcentrators show elevated temperature, whereas the surrounding substrate areas are unheated. Most temperature drops occur across the substrate cutouts at the preconcentrator edges, showing the effectiveness of the thermal isolation provided by these cutouts. The thermistors, located outside the sorbent chambers and near the preconcentrator edges, show 13–33% lower temperatures than the corresponding sorbent chambers.

The areal distribution of temperature within each preconcentrator sorbent chamber is shown in Fig. 3d as a variation from its mean temperature. In this work, a region is considered uniformly heated if the temperature of every location within it is within ±10% of the mean temperature of the region (referenced to the ambient temperature). Based on this definition, the uniformly heated area fraction (*A*_90_) is computed in Fig. 3e.

The simulation results show that the MPCA2 tailored preconcentrator heaters provide highly uniform heating (Fig. 3c–e). Based on the obtained *A*_90_ values, 85.8% of Preconcentrator1, 98.8% of Preconcentrator2, and 99.9% of Preconcentrator3 are uniformly heated. In comparison, the untailored designs using serpentine traces with fixed width and spacing result in nonuniform heating, with the uniformly heated areal fraction being only 62.7% in Preconcentrator1, 33.1% in Preconcentrator2, and 96.9% in Preconcentrator3 (Fig. 3e). Among these, Preconcentrator3 has the least difference in the heating uniformity between the tailored and untailored design, because its small size and simple shape coincidentally permit an untailored heater design to produce good heating uniformity^9^. In contrast, Preconcentrator2 shows the most improvement from the tailored design. Its solid connections to the rest of the chip are relatively large compared to its sorbent chamber, dissipating a significant amount of heat. The tailored design provides more heat near these connections, thus achieving significantly more uniform temperature for the sorbent chamber.

In addition to the preconcentrators, the separation columns, detectors, and carrier gas filter also use tailored heater patterns to compensate for cold spots (section S1), perimeter cutouts for thermal isolation, and closed-loop control for heating. The resulting improvements allow the heating speed for the separation columns to be increased. The details are described in the section S1.

#### AiPD fence electrode

In a PID, a fence electrode located between the anode and cathode is intended to intercept stray current along the ionization chamber surface without affecting the sensing current through the air. In commercial PIDs, the anode and cathode are typically two parallel plates, between which the fence electrode is configured as an annular ring with a larger diameter than the anode or cathode, and recessed into the walls of the ionization chamber. The fence electrode voltage is biased at the nominal cathode voltage. In this configuration, the surface current into the cathode is minimized, while the electric field between the anode and cathode remains almost unaffected by the fence electrode^36^.

**Fig. 4:**
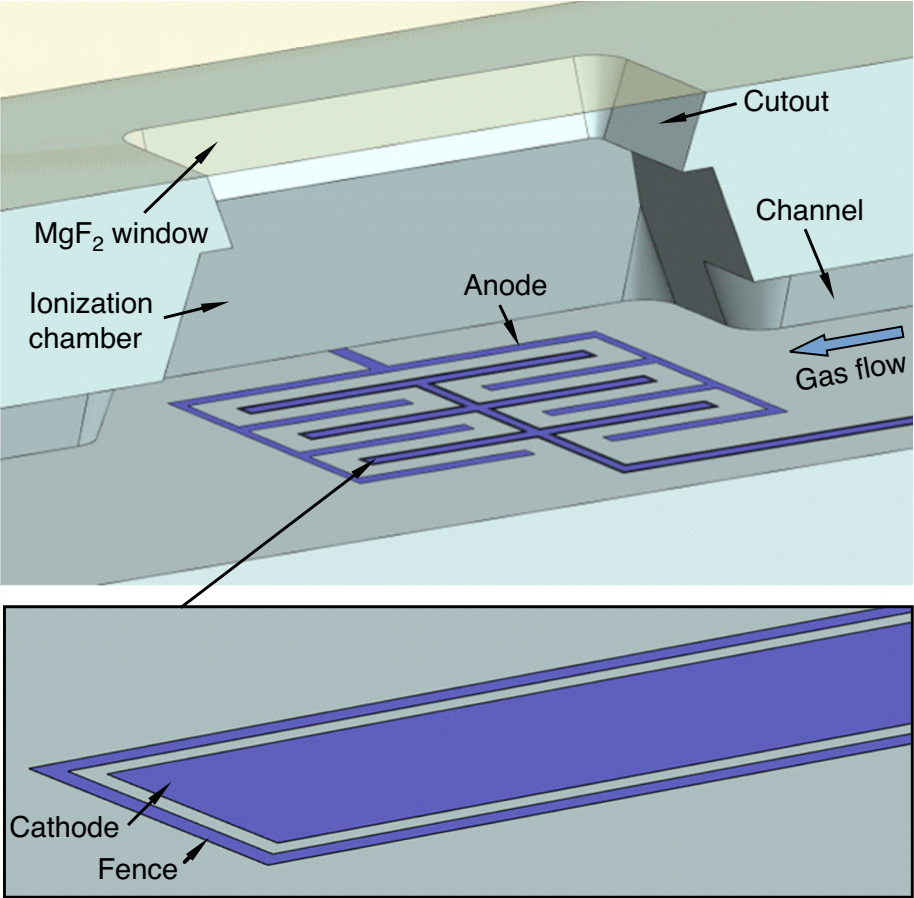
Fence electrode design on the MPCA2 AiPD. The inset shows the fence electrode closely surrounding the cathode

On the MPCA2 chip, all the electrodes are in the form of a Ti/Pt thin film. Therefore, the fence electrode must comply with this provision. To intercept surface current, the fence electrode must form a continuous trace that isolates the anode from the cathode. Additionally, with the fence electrode biased at the nominal cathode voltage, which is below the anode voltage, the fence electrode inevitably affects the electric field between the anode and the cathode and shunts a fraction of the sensing current. To minimize this shunting effect, the fence electrode can be designed as a narrow trace that closely surrounds a much wider cathode trace. With these considerations, in the MPCA2 AiPD, the anode and cathode are designed as interdigitated electrodes with ≈500 μm tine lengths, 50 μm tine widths, and 100 µm tine gaps. In contrast, the fence electrode is designed as a 5 μm wide continuous trace that closely surrounds all the tines of the cathode (with a 5 μm gap) (Fig. 4). With such dimensions, the fringe electric field between the anode and the cathode is largely undisturbed by the fence electrode. The anode consists of 8 tines and the cathode consists of 6 tines; they occupy a sensing area of ≈1 mm^2^.

To benchmark the effectiveness of the fence electrode design, AiPDs without fence electrodes are also included on the chip and positioned next to the MPCA2 AiPDs. Each benchmarking AiPD adopts a previously reported design, which includes interdigitated anode and cathode with 10 µm tine width, 100 µm tine gap, and occupying ≈1 mm^2^ sensing area^9^. For comparison, the anode-cathode gap is kept consistent between the two designs.

### System architecture and enhancements

The full system is assembled in a 30 × 25 × 12 cm^3^ modified weather-resistant commercial enclosure (lm201709071393, LeMotech, Shenzhen, China) (Fig. 2c). For logistics reasons, two copies of the system are built and tested.

#### Fluidic control

A sampling pump (#NMP03KPDC-L, KNF Neuberger Inc., NJ, USA) is used to provide a fixed sampling flow through the preconcentrators at ≈20 sccm. The sampling duration is set by the user. The separation flow is provided by two separation pumps (#mp6-air, Servoflo Corp., MA, USA), one located upstream (i.e., Separation pump 1) to push a flow through the preconcentrators (in the reverse direction of the sample flow) and the other (i.e., Separation pump 2) located downstream to pull a flow through the columns and detectors. By balancing the flows from these two separation pumps, the flow at the sample inlet can be minimized, allowing the elimination of the commonly used valve at the sample inlet that may interfere with the sample. Operationally, the separation flow is set to 0.6 sccm for the entire Cell1 separation, before 260 s of Cell2 separation, and before 150 s of Cell3 separation. After 260 s in Cell2 and after 150 s in Cell3, the separation flow is increased to 1.3 sccm to accelerate the elution of slow-eluting analytes (in addition to the temperature programming as described later). The system also incorporates six solenoid latching valves (#LHLA1231211H, The Lee Co., CT, USA): Valve6 selects between the sampling and separation flow; Valve1, Valve2, and Valve3 each routes the separation flow through its cell, one at a time; and Valve4 and Valve5 may release vapor from the internal reference into the sampling flow path by opening for 10 s during sampling (Fig. 1).

To enhance repeatability in the analyte retention times, closed-loop control of the separation flow rate is implemented. Due to a lack of commercial low-cost, high-resolution flow sensors, differential pressure sensors (#MPX5010DP-ND, Digi-Key^®^ Electronics, MN, USA) are used. The upstream pressure head, which is effectively applied across the preconcentrator in the active cell as well as all the upstream preconcentrators and carrier gas filter, is measured at the outlet of Separation pump 1. The downstream pressure, which is effectively applied across the column and detectors in the active cell, is measured at the inlet of the Separation pump 2. Both pressure values are measured at a rate of 2.5 Hz. The measurements are fed into a proportional-integral-derivative control algorithm, which appropriately adjusts the actuation frequencies of both pumps. Because of the slight differences in the flow resistances and target flow profiles in each cell, the target upstream pressure head and downstream pressure head are also set differently for each cell.

A typical analytical run consists of four steps: conditioning, sampling, purging, and separation (with concurrent detection). Conditioning purges preconcentrators, removing residual analytes from previous runs. During conditioning, preconcentrators are heated to 160 °C for 40 s, while Separation pump 1 pushes 3 sccm flow through all the preconcentrators and out of the sample inlet. In the last 45 s of sampling, the carrier gas filter is desorbed at 160 °C; during this period, Precon1 is heated to 45 °C to desorb water, according to a “warm trap” method reported to reduce water adsorption in carbon molecular sieves during vapor sampling^37^. The third step is a “dry purge” technique^38,39^. In this step, the preconcentrators are purged with a 3 sccm flow from Separation pump 1, then the columns are purged with a flow from both separation pumps for an additional 20 s. All the purge flows generated by the separation pump(s) are ambient air flows, which are dehumidified when passing through the carrier gas filter.

In the separation step, the preconcentrator of each cell is heated for 2–8 s (depending on the cell and the corresponding heating start time) to reach a target temperature of 160 °C at 20 s, maintaining that temperature for 15 s while desorbing the trapped analytes. Higher desorption temperatures may be suitable for future studies. The separation pumps are turned on at 20 s, with the flow direction reversed from sampling. These two component operations combine to effectively set the separation start time for each cell at 20 s. During the separation step, Column 1 is maintained at room temperature, Column 2 is ramped from 30 °C to 60 °C over *t* = 20 s to *t* = 400 s, and Column 3 is ramped from 50 °C to 70 °C, from *t* = 20 s to *t* = 200 s. The programmed column temperatures are set to be modest values to limit the possibility of PDMS degradation^40^ because air is used as the carrier gas. The detectors are maintained at room temperature during Cell1 separation, 45 °C during Cell2 separation, and 65 °C during Cell3 separation.

#### Internal chemical reference

To provide a retention time reference, the internal chemical reference emits a known chemical vapor into the collected sample. In this work, the reference consists of a 1.5 mL vial filled with 0.72 g of a PDMS gum-like sorbent (OV-1, #6001, Ohio Valley Specialty Company, OH, USA), and 1.3 μL o-xylene (Fig. 2d). Here, o-xylene is selected as the reference chemical because it is distributed in both Cell2 and Cell3, providing retention time references for both cells. Depending on the actual applications, another chemical or mixture may be used. When not in operation, the headspace of the reference is sealed by the closed Valve 4 and Valve 5. To release the reference vapor into the sample, Valve 4 and Valve 5 are opened for a short duration, allowing a stream of the sampling flow to carry the vapor into the preconcentrators. During the sampling step, because the gas flow path through the internal reference is parallel to the sample inlet path, the flow rate through the internal reference may be smaller than the nominal 20 sccm sampling flow. As the reference chemical is consumed in each run, with a fixed sampling time for the reference vapor, both the headspace concentration and the resulting peak heights of the reference chemical gradually decrease over the runs. In a first-order calculation neglecting temperature and other variations (section S2), the reference can support ≈2200 runs before 90% of the stored o-xylene is consumed.

## Experimental results and discussion

### AiPD humidity response with fence electrode

To evaluate the effectiveness of the fence electrode, chromatograms obtained from the MPCA2 AiPD and the benchmarking AiPD were compared. These chromatograms were obtained in repeated runs with a consistent set of vapor samples, which contained benzene, toluene, ethylbenzene, m-, p-, and o-xylene (BTEX) at 100 ppb, and three relative humidity (RH) levels at 0, 50%, and 90%. These runs used 10-min sampling durations with ≈20 sccm sampling flow rate; the internal reference was not used.

**Fig. 5:**
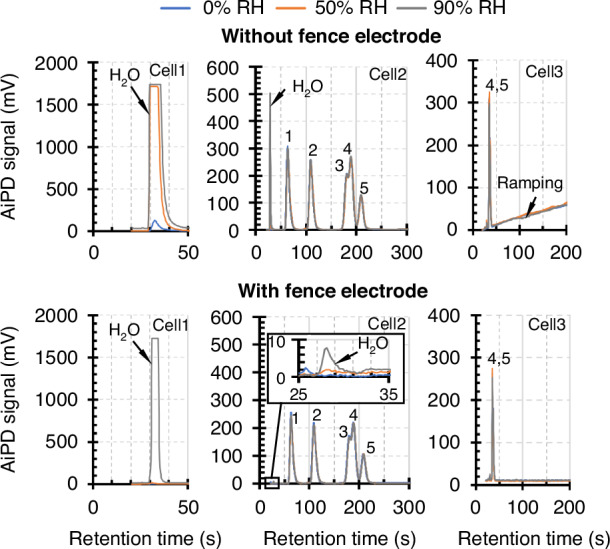
Chromatograms resulting from AiPD designs with and without the fence electrode. Both designs were tested at varying humidity levels with 100 ppb benzene (1), toluene (2), ethylbenzene (3), m/p-xylene (4), and o-xylene (5). In Cell1 and Cell2, the presence of the fence electrode greatly reduces the water peak

The chromatograms from MPCA2 AiPDs (i.e., with the fence electrodes) showed significantly lower humidity responses than the benchmarking AiPDs (with no fence electrode). In Cell2, for the samples with 0, 50%, and 90% RH, the water peak heights from the MPCA2 AiPD were only 0 mV, 2 mV, and 8 mV, respectively (Fig. 5). Compared to the water peak heights of 0 mV, 65 mV, and 503 mV from the benchmarking AiPD for the respective samples, the MPCA2 AiPD reduced the water peak height by >97%.

In Cell1, for the samples with 0 and 50% RH, the water peak heights from the MPCA2 AiPD were only 2.2 mV and 11.9 mV, respectively (Fig. 5), whereas those from the benchmarking AiPD were 109.8 mV and 1700 mV, respectively. Here, the 1700 mV was the upper limit of the readout electronics. For the sample with 90% RH, the water peak in Cell1 from both AiPD designs saturated the readout electronics; nevertheless, the peak width from the MPCA2 AiPD was reduced compared to that from the benchmarking AiPD. In Cell3, the benchmarking AiPD chromatogram showed a baseline drift (of unknown cause) ramping up at 0.32 mV/s; this ramping was not present in the MPCA2 AiPD chromatogram. With respect to the responses to analytes, the BTEX peak heights from the MPCA2 AiPD were lower than those from the benchmarking AiPD by 11–20% (17% on average).

The water peaks in all cases were attributed to water injection by the preconcentrators. Among the three preconcentrators, Precon1 was the most adsorptive and trapped the highest amount of water, Precon2 was moderately adsorptive and trapped a moderate amount of water, whereas Precon3 was the least adsorptive and barely trapped any water. The trapped water was desorbed along with the analytes during the start of the separation, and was quickly eluted from the columns into the detectors.

While the MPCA2 AiPD showed significantly better water rejection than the benchmarking AiPD, the remaining humidity response in the MPCA2 AiPD most likely resulted from the existing coplanar design that did not completely block stray surface currents along the sidewalls of the detector chamber. Such stray currents may be further blocked by improved designs of the electrodes and the chambers in the future. Compared to the benchmarking AiPD, the 11–20% lower analyte sensitivity in the MPCA2 AiPD was likely caused by its fence electrode shunting a portion of the sensing current. For what this feature provides, this reduced sensitivity is acceptable, particularly for the detection of early eluting analytes in the cells.

Certain points are worth noting at the cellular level. Because Precon1 was the most adsorptive, it released the largest quantity of moisture; consequently, the tail of the water peak extended to 90 s (as evident in the CapDet chromatograms in Fig. S3.1), affecting acetone and 2-butanone peaks that eluted closely after the water peak. In contrast, for the AiPD that incorporated the fence electrode, the water peak was suppressed, resulting in more discernible peaks for these analytes in Cell1 (Fig. S3.1). In Cell2 and Cell3, the benefit of the fence electrode was perhaps less evident, as the water peak widths were already small because of the other humidity reduction measures in place, such as the use of nonpolar sorbents and stationary phase, detector heating, and dry purge operation. Nevertheless, for any future permutations that require more polar sorbents and stationary phases, the water bolus may be much wider, and consequently, the benefit of suppressing water responses in the AiPDs may be more pronounced.

### Repeatability and internal reference

The contribution of closed-loop flow control on the retention time repeatability was evaluated by two sets of system runs. The first set was performed using an MPCA system (which was the prior generation system^9^ that did not implement closed-loop flow control). This set incorporated 80 runs with 10-min sampling durations against 20 ppb BTEX. This set was also used to obtain a preliminary indication of false alarm characteristics, as described in the section “False alarm test.” The second set was performed using an MPCA2 system (with closed-loop flow control) and incorporated 70 runs with various sampling durations and against various samples.

It is worth noting that within each cell of the MPCA2 system, the three detectors are arranged in the sequence of CapDetA, CapDetB, and AiPD in the direction of the separation flow. Therefore, when an analyte peak passes through the detectors, there is a delay in the apparent retention times from an upstream detector to a downstream one. Based on the experimental results, the delays from CapDetA to CapDetB were up to 1.5 s in Cell2 and up to 3 s in Cell3, whereas the delays from CapDetB to AiPD were up to 1 s in both Cell2 and Cell3. The delays in Cell1 were not characterized. The retention times reported in this work are mostly from the AiPD responses. Only when a peak has no AiPD response is its retention time reported from the CapDetA response.

**Fig. 6:**
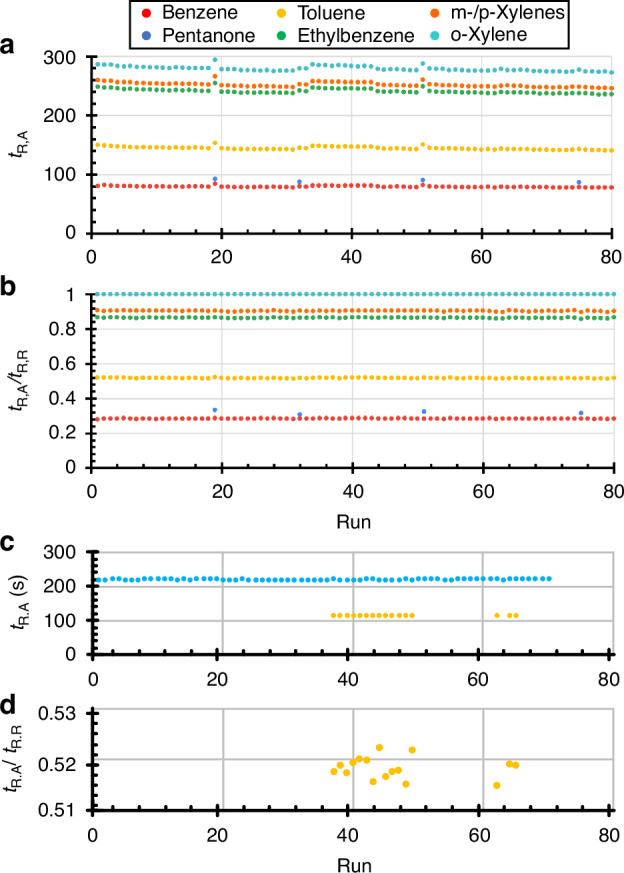
Retention times of analytes from the repeatability test. All the runs used temperature-programmed separations. **a** Raw retention times (*t*_*R.A*_) of the analytes and **b** relative retention times, defined as the retention time ratios between the analyte and the reference (o-xylene) (*t*_*R.A*_*/t*_*R.R*_), from the MPCA system over 80 runs. **c** Raw retention times of toluene and o-xylene from the MPCA2 system over 16 and 70 runs, respectively. **d** Relative retention times of toluene (relative to o-xylene) from the MPCA2 system over 16 runs, the *y*-axis is zoomed into a small range of 0.51–0.53, showing that the data points are tightly distributed

After the first set of runs, the raw retention times (*t*_*R.A*_) were extracted from the chromatograms (Fig. 6a). The relative standard deviation (RSD), i.e., the standard deviation divided by the average, was computed for the *t*_*R.A*_ sample of each chemical. As a result, benzene, toluene, ethylbenzene, m-/p-xylenes, and o-xylene showed RSD values of 1.67, 1.72, 1.53, 1.52, and 1.44%, respectively. The main source of this RSD appeared to be certain shared system variations rather than random noise (i.e., when one analyte had a larger retention time, all the others in that run also had larger retention times).

To investigate the applicability of using a reference chemical for reducing the RSD in the retention time, the relative retention time of a given analyte was computed as the retention time ratio between itself (*t*_*R.A*_) and a reference chemical (*t*_*R.R*_) from the same run^3^. Here, o-xylene was assumed to be the reference chemical. The relative retention times (Fig. 6b) showed RSD values in the range of 0.25–0.36%, which were reduced by a factor of 5.5 on average compared to the RSD values of the raw retention times.

In the MPCA2 system that implemented closed-loop flow control, the internal reference (emitting o-xylene) was used in 70 runs for various tests (Fig. S5.1). Over these 70 runs, the raw retention time of o-xylene showed an RSD of only 0.29% (Fig. 6c), which was ≈5× reduction compared to the prior MPCA system that implemented open-loop flow control. Because toluene was present at non-overloading concentrations in 16 out of these 70 runs, its retention time variation was also worth analyzing (Fig. 6d). Interestingly, toluene had an RSD of 0.43% in the raw retention time and a similar 0.41% in the relative retention time (referencing o-xylene); additionally, these RSD values were similar to those for the relative retention times in the MPCA system. This observation suggests that the RSD in the MPCA system mainly resulted from the carrier gas flow rate variation, which can be effectively suppressed by either the closed-loop flow control or the use of the relative retention time; nevertheless, the combined use of these two methods does not further improve the repeatability in the retention time.

**Fig. 7:**
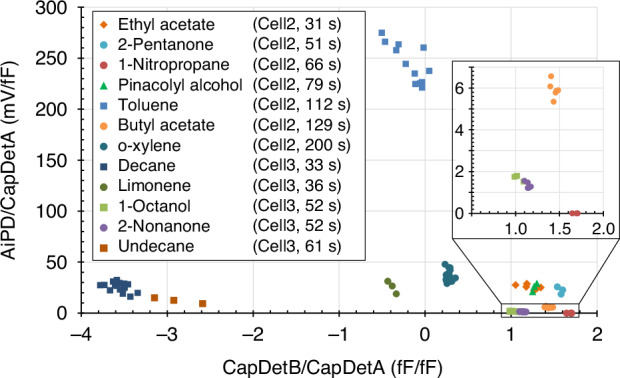
Ratios of peak responses that constitute the DRPs for a portion of the tested analytes. These ratios were extracted from 6 runs containing ethyl acetate, butyl acetate, and 2-nonanone, 3 runs containing pinacolyl alcohol and 1-octanol, another 3 runs containing 2-pentanone, 1-nitropropane, and limonene, another 3 runs containing undecane, 12 runs containing toluene, 16 runs containing decane, and 56 runs containing o-xylene

A study of the experimentally obtained DRPs also revealed several findings. For each analyte, the peak heights obtained from the three detectors were extracted, from which the CapDetB/CapDetA and AiPD/CapDetA ratios were calculated as the DRP (Fig. 7). As evident from Fig. 7, many of the analytes show DRPs that are easily distinguishable from the other analytes, which is useful for recognizing individual chromatographic peaks that are not affected by coelution. For example, the closely eluting decane and limonene show distinctive DRP locations in Fig. 7, indicating that they can be easily differentiated by DRP. An exception pair was ethyl acetate and pinacolyl alcohol, which were indistinguishable by DRP but clearly distinguishable by retention time. A more challenging pair was 1-octanol and 2-nonanone, which had the same retention time and very similar DRPs; future work is needed to investigate how to distinguish these reliably.

### False alarm test

The retention time repeatability test was performed on the MPCA system in a way to also provide preliminary data on false alarms. As such, it was limited in scope: (1) fixed target and interferent analytes were selected to be partially coeluting species but with different DRPs, presenting a moderate level of challenge for recognition; (2) the analyte identities were relatively well known; in other words, unknown analytes were not intentionally included in this test but could be present because of inadvertent contamination present in the ambient, sample preparation, or test setup; (3) a relatively small number of runs were performed.

In this test, an MPCA system was operated for 80 runs against control or spiked samples. As noted previously, the MPCA system did not have improvements in the heaters, fence electrodes, closed-loop flow control, and internal reference. In this test, the control samples contained ≈20 ppb BTEX interferents and 50% RH, whereas spiked samples contained an additional target chemical, 2-pentanone, at ≈20 ppb. Among the interferents, benzene was expected to provide the strongest interference with 2-pentanone, because they would partially coelute based on prior calibration results.

The test was performed as a blind test, separating the operator and the analyst. The operator arranged the number and sequence for the control and spiked samples, prepared the samples, and performed the experiments. The analyst evaluated the experimentally generated chromatograms (e.g., Fig. S4.1) to identify which runs contained the target analyte. The analyst had access to a library of calibration data of the system, but no knowledge of the test conditions used to experimentally generate the chromatograms.

To evaluate each chromatogram, the analyst extracted features from each peak, including the retention times and heights of peaks. Then, chemical recognition was performed based on the retention times and the DRP. Lacking statistics of retention time precision, the analyst set a relatively large retention time window of ±15% around the nominal retention time of the target. Both benzene and 2-pentanone fell within this retention time window, so the distinction between them was based on the DRP. More particularly, the analyst relied on the CapDet peak heights—whether the CapDetB peak was positive and taller than the CapDetA peak; if yes, this peak was polar, representing the 2-pentanone target. (This qualitative rule works well if the 2-pentanone concentration is not too much smaller than the benzene concentration; otherwise, a more quantitative assessment would be needed.)

The analyst identified 4 runs (#19, 32, 51, 74) as those containing the target, and identified all other runs as not containing the target. The operator then revealed the true sample conditions and confirmed that the analyst’s results were correct. In this particular test case, there were no false alarms. A statistical determination of the false alarm rate requires more extensive tests with contextually relevant species^41^; as such, it is beyond the scope of this work.

### Blind chemical recognition tests

#### Test protocol

The MPCA2 systems were tested by a third-party Test and Evaluation (T&E) team at the US Naval Research Laboratory (NRL), as appointed by the sponsor. Among all the tests, one interesting set of tests was a set of blind chemical recognition tests. In these tests, the MPCA2 system was tested against eight separate mixtures that contained various chemicals from a known list of 200 target chemicals. The mixtures were generated using a custom setup^42^ and were verified by a benchtop GC-MS operated by the T&E staff from NRL. To minimize carryover, blank runs (in which the samples were zero air) were inserted by the T&E team after each test. As a blind test, the identities of the chemical instances under test were not revealed to our team until after our team analyzed the chromatograms and submitted the recognition results. Here, the term “instance” refers to the presence of an intentionally introduced test chemical in a run; the same chemical, if appearing again in a different run, constituted a different instance. The MPCA2 system was set for a 15-min sampling duration for this test.

**Fig. 8:**
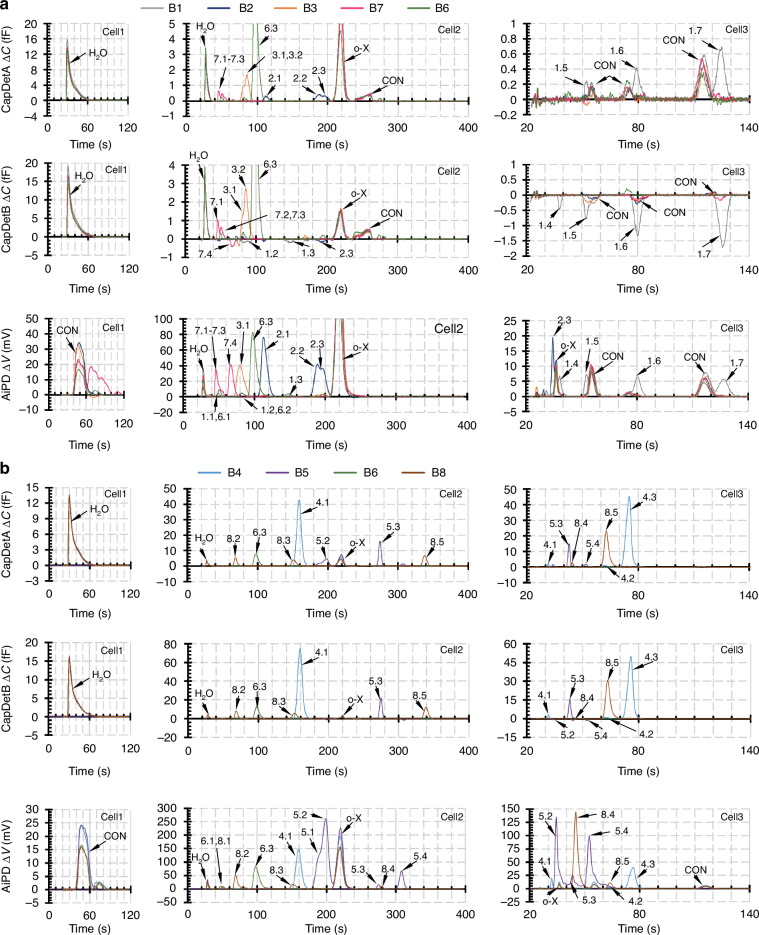
Overlaid chromatograms from the blind chemical recognition test. For visualization, the runs are separately plotted in two groups: **a** runs with relatively small peak heights and **b** runs with relatively large peak heights. Run B6 is shared across both subfigures as it contains both large and small peak heights. Analytes are labeled in paired numbers, representing run number and assigned analyte number within the run. CON refers to contaminants. Corresponding recognition results are summarized in Table 2

To recognize chromatographic peaks from the chromatograms (Fig. 8), our team used a combination of the retention time and DRP. The procedure is described in detail in the following subsection. Due to logistical reasons, at the time of the test, the system had only been partially calibrated (for a subset of 17% of the possible targets). Here, the term “calibrated” refers to at least one run with the known analyte, providing nominal values of the retention time and DRP. Despite the absence of calibration runs for most of the target analytes, both calibrated analytes and uncalibrated analytes were included as possible outcomes in the recognition step.

In this test, up to four candidates were reported for a retention peak, in the order of decreasing probability. The count of possible candidates took into account several factors: (i) the intended use of the instrument as a screening tool; (ii) the large number of possible analytes in the target list; and (iii) the absence of calibration data for most of the analytes. Following the submission of the recognition results, the true chemical identities (i.e., answer keys) were revealed by the T&E team, although the true sample concentrations were not revealed. The 8 runs included 3–7 target chemical instances per run and 28 instances in total; these included single and repeated instances from the 200-chemical target list. The 8 runs also included 3 unlisted chemical instances that were outside the target list. The results are summarized in Table 2. Interestingly, the fourth candidate was never the correct answer, and the third candidate was the correct answer in only one case. In all other cases, the first or second candidate was the correct answer, indicating that in practical applications with a smaller number of target analytes and with calibration data from all target analytes, the number of candidates may be reduced.

**Table 2:** Summarized results of the blind chemical recognition test

Run	Peak	Target	Actual analyte	Reported analytes	Cell	*t*_*R*_ (s)	Peak heights		
							CA (fF)	CB (fF)	AD (mV)
B1	1.1	Yes	Hexane*	Hexane*	2	48.7	≈0	≈0	4.46
	1.2	Yes	Heptane*	Heptane*	2	82.5	≈0	−0.21	4.40
	1.3	Yes	Octane*	Octane*	2	147.8	≈0	−0.21	4.07
	1.4	Yes	Nonane*	Nonane*	3	38.2	≈0	−0.47	6.85
	1.5	Yes	Decane*	Decane*	3	52.7	0.25	−0.79	7.33
	1.6	Yes	Undecane*	Undecane*	3	80.6	0.42	−1.33	6.50
	1.7	Yes	Dodecane	**Dodecane**	3	126.8	0.69	−1.71	5.83
B2	2.1	Yes	Toluene*	Toluene*	2	112.9	0.32	−0.12	76.44
	2.2	Yes	Ethylbenzene*	Ethylbenzene*	2	188.7	0.43	−0.19	41.80
	2.3	Yes	m-xylene	**m-/p-xylene**	2	195.8	0.36	−0.12	36.60
	o-X	Yes	o-xylene*	Reference, not reported	2	220.5	6.29	1.61	214.18
B3	Unassigned	Yes	Isooctane	Not reported					
	3.1	Yes	Pinacolone	**Pinacolone**, n-Propyl acetate	2	80.8	0.95	1.56	41.08
	3.2	Yes	1-Nitropropane	**1-Nitropropane**	2	85.4	1.70	2.94	≈0
B4	4.1	No	2-Methyl-2-pentanal	Chlorobenzene	2	159.8	42.83	74.98	146.46
	4.2	N/A	Not present	Acetophenone	3	63.0	1.07	1.21	2.92
	4.3	Yes	1-Nonanal	2-nonanone*, **1-Nonanal**, 1-Octanol, Phenylethyl alcohol	3	76.3	45.15	50.01	40.06
B5	5.1	N/A	Not present	Ethylbenzene*	2	188.3	1.76	≈0	117.30
	5.2	Yes	p-xylene	**m-/p-xylene**	2	198.5	4.79	−1.16	261.14
	o-X	Yes	o-xylene	Reference, not reported	2	220.1	7.36	1.91	227.33
	5.3	No	Chloroheptane	2-Chlorotoluene, 2-Chlorophenol, 6-Methyl-2-heptanone	2	275.0	16.19	22.49	16.47
	5.4	No	3-carene	1,2,4-Trimethylbenzene, 1,4-Dichlorobenzene	3	52.5	1.91	−1.37	100.06
B6	6.1	Yes	sec-Butyl alcohol	Ethyl acetate, n-Butanal, **Sec-butyl alcohol**	2	50.2	0.23	0.31	9.38
	6.2	N/A	Not present	n-Propyl acetate, Pinacolone, Acetic anhyrdide	2	82.4	0.23	0.37	4.66
	6.3	Yes	Pinacolyl alcohol* and Methyl isobutyl ketone	Pinacolyl alcohol*, Pyridine, Dibromoethane, **Methyl isobutyl ketone**	2	97.6	7.78	12.70	82.94
B7	7.1	Yes	Butanone	2,3-Butanedione, **Butanone**, Vinyl acetate, Methyl tert-butyl ether	2	46.1	0.66	1.06	35.62
	7.2	Yes	Ethyl acetate*	Ethyl acetate*, n-Butanal, sec-Butyl alcohol	2	50.7	0.46	0.65	13.69
	7.3	Yes	Tetrahydrofuran*	Tetrahydrofuran*, Ethyl tert-butyl ether	2	54.8	0.18	≈0	7.67
	7.4	Yes	Cyclohexane*	Cyclohexane*	2	66.5	0.09	−0.41	41.44
B8	8.1	N/A	Not present	sec-Butyl alcohol, n-Butanal, 2-Butanone	2	47.6	0.17	0.27	10.53
	8.2	Yes	1-Butanol*	1-Butanol*, 3-Methyl butanone, 2-Pentanone	2	68.7	5.50	8.17	51.86
	8.3	Yes	Butyl acetate*	Butyl acetate*	2	151.5	4.13	5.96	17.73
	8.4	Yes	Mesitylene*	Mesitylene*	3	45.0	1.6	−1.17	139.37
	8.5	Yes	Nitrobenzene	**Nitrobenzene**	3	63.6	23.10	30.76	10.78

The * indicates the system was calibrated for the listed analyte prior to the test. Up to four candidates are provided in the order of decreasing probability

Correct answers are bolded

*CA* CapDetA, *CB* CapDetB, *AD* AiPD

#### Chemical recognition procedure

Amongst all the peaks within the chromatograms, those that appeared consistently even in the blank runs were treated as contaminants, and no attempt was made to recognize them. These included the CapDet peaks at 240–260 s in Cell2, at 55, 74, 80, and at 115 s in Cell3, and the AiPD peak at 50 s in Cell1. Without the use of a mass spectrometer, it was not possible to determine their identities. These peaks possibly resulted from system outgassing or the background in the test setup. Their impacts were deemed small because of their relatively clear separation from other analytes.

The remaining peaks were treated as resulting from an analyte, and the following recognition procedure was performed. First, the measured retention time and the estimated Kovats retention index (RI, which is a unitless number) were compared against the calibrated or expected values of the chemicals from the target list. For a calibrated target analyte, if its calibrated retention time was within approximately ±5% of the measured retention time, it was considered a match in retention time and flagged as a potential candidate for the retention peak. Compared to the RSD variation of <0.5% (as presented in the section “Blind chemical recognition tests”), this larger tolerance ±5% was set because this tolerance should cover the peak-to-peak variation rather than RSD, with some additional tolerance. For an uncalibrated chemical, if its RI differed from the estimated RI of the peak by less than ±20, it was also considered a match in retention time and flagged as a potential candidate, albeit with a lower confidence level. This tolerance was much larger, mainly due to uncertainties and variations in the estimated RI values as determined from online databases.

For each retention peak, the second recognition step was to compare its measured DRP against those of the potential candidates selected on the basis of retention time alone. In the DRP comparison, the CapDetA/CapDetB ratio was reliable for categorizing the analyte polarity. This ratio ranged from ~0.5–1 for polar analytes, −1 to 0 for highly nonpolar analytes (e.g., alkanes), and was less than −1 for moderately nonpolar analytes (e.g., BTEX)^9,31^. Therefore, based on the measured CapDetA/CapDetB ratio of the retention peak, its polarity category was first determined, and then compared to those of the candidates identified by retention time. For an uncalibrated candidate, the CapDetA/CapDetB ratio was predicted based on its dielectric constant.

Subsequently, the CapDetA/AiPD and CapDetB/AiPD ratios were further considered. For a calibrated candidate, if the CapDet/AiPD ratio was roughly within ±50% of the measured ratio, it was flagged as a potential match. Accommodations were made for peaks affected by overloading (if the peak height was large with fronting), noise (if the peak height was small), coelution of a neighboring peak, and AiPD non-linearity^9^. For an uncalibrated candidate, its CapDetA/AiPD and CapDetB/AiPD ratios were coarsely projected from data of other calibration chemicals with similar RI, polarity, and ionization potential values. Then the projected ratios were compared to the measured ratios to provide a determination with a lower confidence level than all previously listed criteria. These general guidelines, in some cases, yielded multiple possible chemicals matching a single retention peak, which were then ordered in the sequence of decreasing probability. This probability ranking was assessed case by case, typically depending on how close the RI and the DRP were between the measured peak and the expected values of the candidate.

The chemical recognition procedure is illustrated through experimental examples. A relatively simple example, Run B8, contained a peak (Peak 8.4) at 45.0 s in Cell3 with a peak height ratio of −1.4 fF/fF for CapDetA/CapDetB, 0.011 fF/mV for CapDetA/AiPD, and −0.008 fF/mV for CapDetB/AiPD. In the target list, mesitylene was a calibrated chemical, nominally in Cell3 at 44.7 s, which was a very good match; additionally, mesitylene had calibrated peak height ratios of −2.2 fF/fF for CapDetA/CapDetB, 0.016 fF/mV for CapDetA/AiPD, and −0.007 fF/mV for CapDetB/AiPD, all considered matching to Peak 8.4. Therefore, mesitylene was reported for Peak 8.4 with high confidence. Additionally, Peak 8.4 was estimated to have an RI of 956, the same as mesitylene. In the target list, there were 6 more analytes that were within the RI range of 956 ± 20: aniline, 6-methyl-2-heptanone, diethylphosphite, 2-chlorophenol, 2-chlorotoluene, and diethyl methyl phosphonate. All these 6 analytes were uncalibrated and were considered potential candidates by RI alone. Coincidentally, all 6 were polar analytes that would not generate a negative CapDetA/CapDetB ratio as Peak 8.4. Therefore, all 6 were excluded from the final recognition result, and mesitylene was submitted as the solitary candidate, which proved to be correct.

A relatively complex example, Run B7, contained three coeluting peaks in Cell2: Peaks 7.1, 7.2, and 7.3 at 46.1 s, 50.7 s, and 54.8 s, respectively (Fig. 8a). The latter peaks appeared as small bumps on the tails of preceding peaks in the AiPD chromatograms. Therefore, without mathematically resolving the coelution, there were large uncertainties in their measured peak heights, particularly in the AiPD chromatogram. Using Peak 7.2 as an example, its retention time at 50.7 s matched well with ethyl acetate, which was a calibrated chemical with a peak expected at 51.1 s. The experimental DRP of 0.71 fF/fF, 0.034 fF/mV, and 0.047 fF/mV also matched well with the calibrated DRP for ethyl acetate at 0.90 fF/fF, 0.038 fF/mV, and 0.042 fF/mV, respectively. Within the RI range of 602 ± 20, there were 12 more potential candidates, including the hexane and tetrahydrofuran (both calibrated prior to the test), and uncalibrated 2-butanone (which was not calibrated at the time of the test), acetic acid, n-butanal, sec-butyl alcohol, chloroform, diisopropyl ether, 2-chloroethanol, nitroethane, ethyl tert-butyl ether, and 1,2-dichloroethane. Hexane was easily ruled out because it was highly nonpolar and would not generate the positive CapDetB response in Peak 7.2. Acetic acid, chloroform, 2-chloroethanol, nitroethane, and 1,2-dichloroethane were also easily ruled out, because they have ionization potentials above 10.6 eV and would not generate the AiPD response observed in Peak 7.2. Diisopropyl ether was ruled out because of its much lower ionization potential at 9.2 eV, which was expected to generate a much larger AiPD response than that in Peak 7.2. Tetrahydrofuran, ethyl tert-butyl ether, and 2-butanone were ruled out, both because their RIs differed from the estimated 602 by ≥15 and because their ionization potentials were low (9.39–9.54). The remaining n-butanal and sec-butyl alcohol have very similar RIs, ionization potentials, and polarity category as ethyl acetate and were not ruled out. Ultimately, ethyl acetate was reported as the primary candidate, whereas n-butanal and sec-butyl alcohol were reported as the second and third. Peak 7.2 turned out to be ethyl acetate.

#### Recognition results and discussion

Out of the 28 chemical instances that were from the target list, 25 instances were correctly recognized by reporting a total of 43 candidates. An instance of isooctane was missed. The other two instances that were missed were both o-xylene, which coincided with the choice for the reference chemical and was consequently excluded from the recognition procedure. There were chemicals that were included in the test but were not included in the target list (3-carene, 2-methyl-2-pentanal, chloroheptane); these were misrecognized as other target chemicals.

For certain chemical families, the enhancement in recognition afforded by the DRP was particularly significant. Analytes with ionization potentials >10.6 eV could be easily distinguished from those <10.6 eV, based on the presence or absence of an AiPD response. For example, 1-nitropropane is polar and has an ionization potential at 10.81 eV. It was uniquely and correctly reported for Peak 3.2, among 7 other uncalibrated polar analytes with similar RIs (within difference of ±20). Most nonpolar compounds were uniquely recognized because of their negative CapDetB responses. Particularly, Peak 7.4 in Run 7 was uniquely recognized as cyclohexane. It is well known that cyclohexane and benzene interfere with each other in GC; benzene is a common target, being a carcinogen that is regulated by many agencies. In this test, benzene was also in the target list and was correctly excluded from the recognition result for cyclohexane.

Ignoring the o-xylene (which was coincidentally also the reference chemical), the only missed case for nonpolar compounds was isooctane, which coeluted with another correctly recognized analyte (pinacolone). In fact, there was a small negative CapDetB peak immediately prior to Peak 3.1 in the chromatogram that very likely corresponded with isooctane, which is known to be nonpolar. Unfortunately, this negative peak was neglected by our team during the recognition exercise, so this miss could be partly attributable to human error.

There were 4 peaks (Peaks 4.2, 5.1, 6.2, 8.1) where none of the reported candidates matched the true identities. Amongst these, Peak 5.1 largely coeluted with m-/p-xylene, potentially causing inaccuracies in the extraction of retention time and DRP information from the peak. Despite such inaccuracies, it was enough to deduce that Peak 5.1 resulted from a nonpolar chemical with a low ionization potential that was eluting immediately before m-/p-xylene. These signs led to the recognition of Peak 5.1 as ethylbenzene. However, there was no chemical in the answer keys that corresponded to this peak, indicating a possible error in the answer keys. Peaks 4.2, 6.2, and 8.1 were misrecognized; these peaks had relatively low peak heights (which were <11 mV in the AiPD and <0.5 fF in the CapDets) and likely resulted from the presence of low concentrations of impurities in the test samples.

There were three peaks resulting from chemicals that were outside of the target list: Peak 4.1 from 2-methyl-2-pentanal, Peak 5.3 from chloroheptane, and Peak 5.4 from 3-carene. Unsurprisingly, these peaks were recognized as other chemicals that were within the target list; the true identities were revealed only by the answer keys afterwards. Although it was impossible for the system to recognize unlisted chemicals, some incorrect candidates for these peaks could have been avoided by calibration with more chemicals and by more careful analysis of the DRP. For example, it was an apparent mistake to choose chlorotoluene as a candidate for Peak 5.3, which had an AiPD response that was considerably lower than those that would be provided by halogenated aromatics (typically with low ionization energies associated with the benzene ring structure).

The recognition accuracy can be further improved by calibration of more chemicals, allowing the use of calibrated retention times rather than coarsely estimated RIs, and allowing the DRP to resolve such fully coeluting pairs^8,31,43^ and screen out unlikely candidates. Additionally, the retention time variation data (Fig. 6) and DRP variation data (Fig. 7), which were only available after the test, can be used to improve the recognition in the future. Among the four improvements reported for MPCA2, the closed-loop flow control and the internal reference were particularly important for this chemical recognition test. The closed-loop flow control provides sufficient repeatability in the retention time, allowing the use of relatively tight tolerances in the retention time for calibrated chemicals. The internal reference served as a good verification of the retention time repeatability. In the future, as we are now more confident with the retention time repeatability, the internal reference may be disabled for most analytical runs and only used as needed, e.g., in occasional calibration or verification runs, or in environments with varied ambient pressures. Additionally, the internal reference may also be used to check for DRP drift. Overall, the blind chemical recognition test results showed the promise of the MPCA2 system for broad chemical screening.

## Conclusions

This work investigated avenues to achieving high reliability in chemical analysis for an integrated µGC with an MPCA. For a highly integrated µGC chip on a thermally non-conductive substrate, heating uniformity was achieved by tailored heater designs that were assisted and verified by FEA. For the on-chip PIDs with coplanar electrodes, the incorporation of the fence electrodes significantly suppressed the humidity response. By implementing closed-loop flow control, the retention time variation of the system was only 0.29–0.43%, achieving 5× reduction compared to open-loop flow control. A similar level of reduction was alternatively achieved by the use of relative retention time with respect to a reference chemical, which was emitted by an easily configurable internal reference. A chemical recognition procedure suitable for screening applications was developed using a combination of retention time and DRP, and its effectiveness was assessed in a preliminary blind test. While effective, the recognition can be improved by full calibration of the system, which can provide more accurate retention time and DRP information than the prediction from chemical properties.

Beyond the advancements reported in this paper, the MPCA2 system can be further improved in the future to extend its capabilities. For example, a porous layer coating can be developed for the Cell1 column and CapDets to improve the analysis of highly volatile chemicals. Such a coating may change how the water peak elutes from Cell1, then further measures can be investigated to reduce the impact of humidity in Cell1. The arrangements of Cell2 and Cell3 can be adjusted depending on the application. If analysis of heavier analytes (than the currently demonstrated dodecane) is required, Cell3 can be adjusted to incorporate a weaker sorbent material and a thinner stationary phase. In contrast, if the application only requires detection of chemicals as heavy as nonane or decane, Cell3 can be eliminated while extending the Cell2 heating and analysis duration. These adjustments must also consider the energy budget, if any, and the temperature limit of the stationary phase material while using air as the carrier gas; these topics are out of the scope of this work.

Future development of MPCA will also include building software for automatic peak detection, chemical recognition, and quantification, including using the DRP to resolve fully coeluting peaks. Future work may also include identifying the contaminants resulting from system outgassing, which may lead to further improvements.

## Supplementary information

Supplementary Material
