# Supplementary material for: An enhanced-performance multisensing progressive cellular μGC: design advances and blind test results
Source: Microsyst Nanoeng. 2025 Jul 11;11:141. doi: 10.1038/s41378-025-00984-6 (PMC12254352; doi:10.1038/s41378-025-00984-6)
Supplement: Supplementary file 1 — Supplementary Material [file 41378_2025_984_MOESM1_ESM.pdf]

Supplementary Information

An Enhanced-performance Multisensing Progressive Cellular μGC: Design Advances and Blind Test Results

Declan Winship[1, †], Weilin Liao[1, †], Hsueh-Tsung Lu[2], Irene Lara-Ibeas[1], Xiangyu Zhao[1], Qu Xu[3], Tao Qian[1], Robert Gordenker[1], Yutao Qin[1,*], Yogesh B. Gianchandani[1,2,*]

[1]Department of Electrical Engineering and Computer Science, and Center for Wireless Integrated MicroSensing and Systems (WIMS^2), University of Michigan, Ann Arbor, MI 48109, USA
[2]Department of Mechanical Engineering, and Center for Wireless Integrated MicroSensing and Systems (WIMS^2), University of Michigan, Ann Arbor, MI 48109, USA
[3]Department of Integrative Systems + Design, and Center for Wireless Integrated MicroSensing and Systems (WIMS^2), University of Michigan, Ann Arbor, MI 48109, USA
*Corresponding authors
†Equal contribution

Contents:

S1. Thermal Simulation Logistics
S2. Reference Standard Depletion Rate Analysis
S3. Chemical Testing
S4: False Alarm Test and Repeatability Test Chromatograms
S5: Overlaid AiPD chromatograms

S1. Thermal Simulation Logistics

The thermal conductivities of each simulated material can be found in Table S1.1. The thermal conductivity of the Ti/Pt was measured experimentally, and the sorbent beds were modeled as solid materials [S1]. The fluidic channels and thermal isolation cutouts were modeled as air. The simulated resistances were verified by comparing the experimental MPCA resistances to the simulated MPCA resistances (Table S1.2)

Table S1.1: Thermal conductivity values assumed in FEA

Materials	Thermal conductivity value ($Wm^{-1}K^{-1}$)
Fused silica	1.4
Sorbent material	6.0
PCB	0.30
Ultem 1010	0.22
Ti/Pt	71.6
Air	0.03

Table S1.2: Measured and simulated electrical resistance of each heater at 20°C in MPCA

Heater	Measured resistance (Ω)	Simulated resistance (Ω)
Precon1	85.2	90.5
Precon2	94.2	101.0
Precon3	99.3	117.2
Carrier gas filter	80.0	97.2
Column1	274.0 (average)	288.3
Column2		281.8
Column3		293.7
Detector	166.0	157.9

Even with a non-compensatory heater design, the separation column heaters show relatively uniform heating without the need for tailored traces. However, in an earlier design [S2], the column heater resistances were 282-294 Ω, which limited the heating power to 1.95-2.05 W. To provide a larger heating power for MPCA2 (in case the system needs to operate in a colder environment), a tailored column heater design reduces the heater resistances to 171-181 Ω, allowing ≈33% higher heating power (3.18-3.37 W) while maintaining heating uniformity (Table S1.3, Figure S1.1a).

Table S1.3: Simulated column temperature distribution nonuniformity

	Untailored Heater			Tailored Heater		
	Cell1	Cell2	Cell3	Cell1	Cell2	Cell3
ΔT_{C-P} (°C)	3.0	2.2	4.0	3.1	1.1	3.3
$\Delta T_{C-P}/T_C$ (%)	7.5	5.3	10.2	5.6	1.8	5.6
$r_{T,0}$ (°C/s)	0.80	0.84	0.78	1.11	1.24	1.18
T_C (°C)	61.2	63.2	60.2	74.9	82.8	80.1
V_{90} (%)	0.754	0.734	0.986	0.928	0.977	0.999
T_C: Temperature at the center of the component at steady state; ΔT_{C-P}: Temperature difference between center of the component and the perimeter at steady state; $r_{T,0}$: temperature ramp rate at t = 0 s; V_{90}: fraction of preconcentrator volume which is warmer than $0.9T_c$						

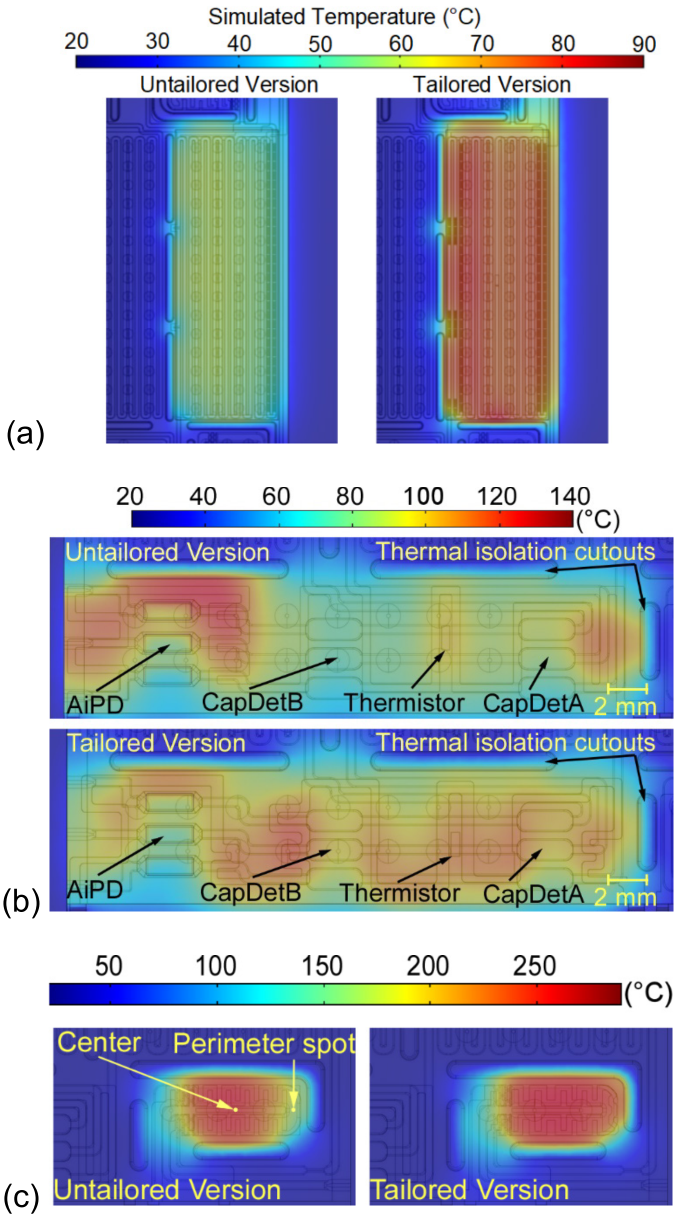

Figure S1.1: Comparison of simulated temperature distributions between untailored and tailored heater design for (a) separation columns, (b) detector heaters and (c) the CGF.

The thermal simulations for the detector heater (Figure S1.1b) show much improved temperature uniformity by using a tailored compensatory heater design. The carrier gas filter (CGF) temperature uniformity was also improved (Figure S1.1c). Like the preconcentrators, increased uniformity for the CGF enhances chemical desorption when the CGF is heated. For the CGF, this allows the trapped water to be more effectively desorbed in the purge steps, thus regenerating the CGF more effectively. The CGF in MPCA2 had a $\Delta T_{C\text{-}P}$ of 9.8°C compared to 27.5°C in MPCA ($\Delta T_{C\text{-}P}/T_C$ was 4.3% in MPCA2 compared to 13.5% in MPCA).

S2. Reference Standard Depletion Rate Analysis

Based on the assumption that the partition equilibrium can be maintained during the sampling step, the depletion rate of the internal reference can be estimated. The fraction of analyte molecules in the air (η) rather than in the sorbent is [S3]:

$$\eta = \frac{1}{1+\frac{K_D}{\beta}} \; ; \; \beta = \frac{V_{air}}{V_{PDMS}} \tag{S2.1}$$

where K_D is the partition coefficient for the analyte between PDMS and air, V_{air} is the volume of air in the internal reference, and V_{PDMS} is the volume of PDMS. The mass of xylene removed from the reference over time (dm/dt) is dependent on the mass of xylene remaining in the reference ($m(t)$), such that:

$$\frac{dm}{dt} = -m(t)\cdot \eta \cdot \frac{Q}{V_{air}} \tag{S2.2}$$

where Q is the volumetric sampling flow rate. Solving Equation S2.2 yields:

$$m(t) = m_0 \cdot e^{\frac{-\eta Qt}{V_{air}}} \tag{S2.3}$$

where m_0 is the initial mass of xylene. Substituting Equation S2.1 into S2.3 yields:

$$m(t) = m_0 \cdot e^{\frac{-Qt}{V_{air}+V_{PDMS}K_D}} \tag{S2.4}$$

For the internal reference, m_0 is 1.144 mg, V_{PDMS} is 0.75 mL, and V_{air} is 1.15 mL. The K_D for o-xylene and PDMS is 3193 [S4]. Defining the lifetime of the reference to be when the o-xylene content is 10% of its original value (0.1144 mg), the lifetime becomes 367 minutes. If the sampling time each run is 10 s with a flow rate of 15 sccm, the o-xylene would last 2208 runs before depleting. The initial calculated headspace is 82 ppm o-xylene, which produces appropriate reference peaks in the detectors (with a signal-to-noise ratio up to 3580).

S3. Chemical Testing

A chromatogram generated by the MPCA2 system is shown in Figure S3.1. For this chromatogram, the analyte vapor was generated by an in-house vapor dilution setup [S2]. Analytes were introduced by a syringe pump into a dilution flow of zero air controlled by a mass flow controller (#1000 sccm, Alicat Scientific, AZ, USA). This setup also provided the capability to adjust the humidity level, by mixing this diluted flow with another air stream that was elevated to 100% relative humidity by a bubbler and controlled by a second mass flow controller (#100 sccm, Alicat Scientific, AZ, USA). Prior to this test, the system was calibrated for the retention time of the 20 analytes using 8 simpler mixtures of analytes with well resolved peaks. Then for the run that produced Figure S3.1, a mixture of 20 analytes (Table S3.1) was prepared at 20 ppb concentration levels, while no humidity was intentionally introduced into the sample.

In this chromatogram (Figure S3.1), the analytes that were relatively well separated from others included acetone and 2-butanone in Cell1; 2-butanone, butyl acetate, o-xylene, nonane, and nitrobenzene in Cell2; and nitrobenzene, 2-nonanone, undecane, and dodecane in Cell3. In Cell2, cyclohexane, pentanal, heptane, pinacolyl alcohol, methyl isobutyl ketone, and toluene showed various degrees of overlapping peaks, and ethylbenzene and m-xylene showed incomplete but non-trivial overlapping peaks. In Cell3, ethylbenzene, m-xylene, o-xylene, and nonane showed various degrees of overlapping peaks; nevertheless, considering that they were

much better separated in Cell2, only their Cell2 peaks would be used in practice. Additionally, in Cell3, 1-chloroheptane and mesitylene showed incomplete but non-trivial overlapping peaks.

In the main paper Section 3.4, some examples are provided of how overlapping peaks from blind tests may be distinguished by using the detector response pattern. A similar approach may be used for the chromatograms in Figure S3.1

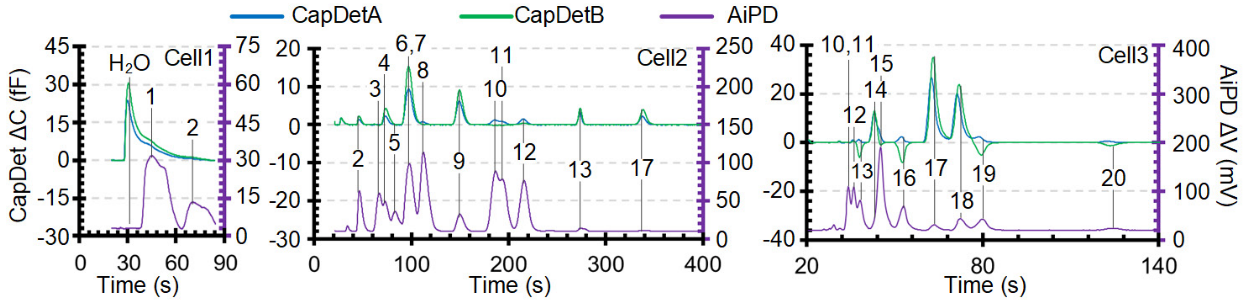

Figure S3.1: Chromatograms of 20 analytes with nominal concentration of 200 ppb generated by the detectors. To facilitate visualization, each sub-chromatogram was processed by applying a moving average and then providing baseline compensation. The moving average used window sizes of 3 s in Cell1, 0.7 s in Cell2, and 0.3 s in Cell3; these values were chosen to preserve the peak heights and widths while reducing noise.

Table S3.1: List of 20 analytes used in the test for Figure S3.1.

Label	Analyte	RI	Molecular Weight (g/mol)	Vapor Pressure at 25°C (mmHg)	ε	IE (eV)
1	Acetone	475	58.1	180*	20.7	9.7
2	2-Butanone	581	72.1	90.6	17.37	9.54
3	Cyclohexane	655.8	84.2	96.9	2.02	9.88
4	Pentanal	674	86.1	26*	10	9.7
5	Heptane	700	100.2	34.5*	1.9	10
6	Pinacolyl Alcohol	721	102.2	8.81	UA	UA
7	Methyl Isobutyl Ketone	729.5	100.2	19.9	13.1	9.3
8	Toluene	755	92.1	28.5*	2.4	8.8
9	Butyl Acetate	804	116.16	11.51	5.1	10
10	Ethylbenzene	854	106.2	7*	2.3	8.8
11	m-Xylene	866	106.2	9*	2.4	8.6
12	o-Xylene	878	106.6	7*	2.6	8.6
13	Nonane	900	128.3	4.4	2.0	10.2
14	1-Chloroheptane	948	134.7	2.82	5.52	10.15
15	Mesitylene	956	120.2	2.48	2.3	8.4
16	Decane	1000	142.3	1.43	2	9.7
17	Nitrobenzene	1049	123.1	0.25	34.9	9.92
18	2-Nonanone	1070	142.2	0.62	9.14	9.16
19	Undecane	1100	156.3	0.41	2	9.5
20	Dodecane	1200	170.3	0.13	2.0	-

RI: Kovats retention index [S5]; ε: Dielectric constant [S6]; IE: Ionization potential [S7]; *Vapor pressure at 20°C [S5], UA: Unavailable

Despite the absence of intentionally introduced humidity in the sample, water peaks were observed in the responses of Cell1 capacitive detectors (Figure S3.1), with relatively large peak heights (up to 30 fF) and tails lasting up to 90 s, affecting the acetone and 2-butanone peaks.

These relatively large water peaks were likely caused by residual moisture in the system, which was not purged prior to these particular tests. Because this humidity response only affects Cell1, its impact is limited.

S4: False Alarm Test and Repeatability Test Chromatograms

Chromatograms from the false alarm test provide insight into the encouraging false alarm test results (Figure S4.1). In the presumed absence of pentanone, the CapDets show very little response to the interferent benzene, while the AiPD shows a strong benzene peak. With pentanone presumed present, the peaks clearly coelute in the AiPD, but the strong response of pentanone in the CapDets significantly changes the detector response pattern. This in turn shows that a different chemical is present in the sample, rather than an elevated quantity of benzene.

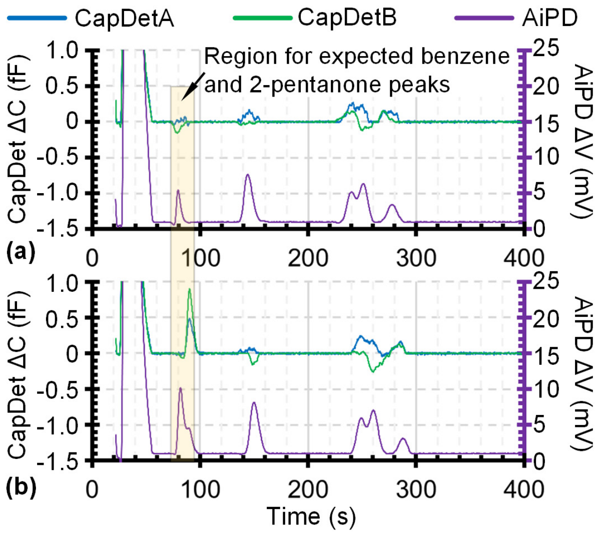

Figure S4.1: Representative chromatograms from Cell2 in two runs during the false alarm test, with (a) the target presumed to be absent, and (b) the target presumed to be present.

S5. Overlaid AiPD chromatograms

Figure S5.1 shows an overlay of AiPD chromatograms over 70 runs. These runs contained o-xylene, which was either emitted from the internal reference, present in the test sample, or carryover from preceding runs. The overlaid o-xylene peaks provide an intuitive visual representation of retention time precision.

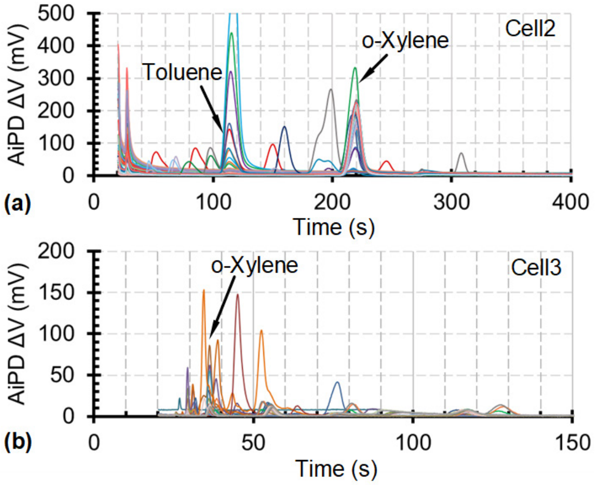

Figure S5.1: Overlaid 70 AiPD chromatograms in (a) Cell2 and (b) Cell3 for a variety of sample mixtures, where o-xylene was present.

References

[S1] A. B. Duncan, G. P. Peterson, and F. S. Fletcher, "Effective Thermal Conductivity Within Packed Beds of Spherical Particles" *Journal of Heat Transfer*, **1989**, *111*, 4, 830-836,

[S2] W. Liao, D. Winship, I. Lara-Ibeas, X. Zhao, Q. Xu, H.-T. Lu, T. Qian, R. Gordenker, Y. Qin, Y. B. Gianchandani, "Highly Integrated µGC Based on a Multisensing Progressive Cellular Architecture with a Valveless Sample Inlet", *Anal. Chem.* **2023**, *95*, 2157–2167

[S3] R.L. Grob, E.F. Barry, *Modern Practice of Gas Chromatography*, John Wiley & Sons, Inc., **2004**.

[S4] A. Kloskowski, W. Chrzanowski, M. Pilarczyk, J. Namieśnik, "Partition coefficients of selected environmetnally important volatile organic compounds determined by gas-liquid chromatography with polydimethylsiloxane stationary phase," *Journal of Chemical Thermaldynamics*, **2005**, *37*, 21-29

[S5] *PubChem Compound Summary for CID 8130*; National Center for Biotechnology Information, 2022, (Online). Available: https://pubchem-ncbi-nlm-nih-gov

[S6] *Springer Materials Interactive*; Springer-Verlag Berlin Heidelberg: Germany, 2017. (Online). Available: https://materials-springer-com /interactive?systemId=8556&propertyId=Dielectric+Constant.

[S7] *NIST Chemistry WebBook*; SRD 69, National Institute of Standards and Technology, 2022. (Online). Available: https://webbook.nist.gov/chemistry
